# A Novel Ferroptosis-Related Gene Signature to Predict Prognosis of Esophageal Carcinoma

**DOI:** 10.1155/2022/7485435

**Published:** 2022-07-01

**Authors:** Jian Wang, Ziming Guo, Fei Sun, Tian Xu, Jianlin Wang, Jingping Yu

**Affiliations:** ^1^Department of Radiotherapy, Jiangyin People's Hospital, Jiangyin 214400, Jiangsu Province, China; ^2^Department of Radiotherapy, The Affiliated Changzhou Second People's Hospital of Nanjing Medical University, Changzhou 213003, Jiangsu Province, China; ^3^Department of Radiotherapy, Shuguang Hospital, Shanghai University of Chinese Traditional Medicine, Zhang Heng Road, Pudong New Area, Shanghai 201203, China

## Abstract

**Objective:**

This study aimed to develop a novel ferroptosis-related gene-based prognostic signature for esophageal carcinoma (ESCA).

**Methods:**

The TCGA-ESCA gene expression profiles and corresponding clinical data were downloaded from the TCGA database. Ferroptosis-related genes were identified from the literature and public databases, which were intersected with the differentially expressed genes between ESCA and normal samples. After univariate Cox regression and random forest analyses, several ferroptosis-related feature genes were identified and used to construct a prognostic signature. Then, the prognostic value of the complex value and the correlation of the complex value with immune cell infiltration were analyzed. Moreover, function analysis, mutation analysis, and molecular docking on the ferroptosis-related feature genes were performed.

**Results:**

Based on the TCGA dataset and ferroptosis pathway genes, 1929 ferroptosis-related genes were preliminarily selected. Following univariate Cox regression analysis and survival analysis, 14 genes were obtained. Then, random forest analysis identified 10 ferroptosis key genes. These 10 genes were used to construct a prognostic complex value. It was found that low complex value indicated better prognosis compared with high complex value. In different ESCA datasets, there were similar differences in the proportion of immune cell distribution between the high and low complex value groups. Furthermore, *TNKS1BP1*, *AC019100.7*, *KRI1*, *BCAP31,* and *RP11-408E5.5* were significantly correlated with ESCA tumor location, lymph node metastasis, and age of patients. *KRI1* had the highest mutation frequency. *BCAP31* had the strongest binding ability with small molecules DB12830, DB05812, and DB07307.

**Conclusion:**

We constructed a novel ferroptosis-related gene signature, which has the potential to predict patient survival and tumor-infiltrating immune cells of ESCA.

## 1. Introduction

Esophageal carcinoma (ESCA) is a malignant digestive system cancer with high morbidity and mortality worldwide [1]. It has two main histopathological subtypes: esophageal adenocarcinoma (EAC), which is common in western countries, and esophageal squamous cell carcinoma (ESCC), which is a dominant pathological type in Asia [2]. Presently, there are several treatment modalities for ESCA, among which surgery and radiotherapy are the most common ones [3, 4]. Thereinto, endoscopic mucosal resection is mainly used for the treatment of precancerous lesions and early ESCA [5]. Nevertheless, ESCA is characterized by a high rate of lymph node metastasis and tumor invasion of adjacent tissues and organs, resulting in a high percentage of patients with metastasis before diagnosis [6]. Due to the metastasis, ESCA patients have a poor prognosis, with a 5-year survival rate of only 18.3% [7]. Because of the limited therapeutic strategies for ESCA, there is an additional need for the development of novel prognostic models.

Ferroptosis is an iron-dependent form of programed cell death, which is driven by accumulation of iron-dependent lipid reactive oxygen species [8]. It is well known that a major hallmark of cancer is its success in evading the regulated forms of cell death [9]. Thus, the induction of ferroptosis has recently emerged as a promising alternative treatment to trigger cancer cell death, especially for malignancies that are resistant to conventional treatments [10, 11]. Many genes have been suggested to promote ferroptosis in cancer cells, such as tumor protein P53 (*TP53*) [12], F-box and WD repeat domain containing 7 (*FBXW7*) [13], and glucose-6-phosphate dehydrogenase (*G6PD*) [8]. DnaJ/Hsp40 homolog subfamily B member 6 (*DNAJB6*) was recently reported to promotes ferroptosis in ESCC [14]. Importantly, Lu et al. [15] reported a ferroptosis-related gene-based prognostic model that independently associated with the overall survival (OS) of ESCC. Song et al. [16] also develop a ferroptosis-related gene-based prognostic signature to predict the OS of ESCC and monitor the immune status. However, most of the studies focused on ESCC.

In the present study, we intended to develop a novel ferroptosis-related gene-based prognostic signature for ESCA based on the gene expression profiles and corresponding clinical data of ESCA patients from public databases. After univariate Cox regression and random forest analyses, we identified several ferroptosis-related feature genes and constructed a prognostic complex value. Then, the prognostic value of the complex value and the correlation of the complex value with immune cell infiltration were analyzed. Moreover, function analysis, mutation analysis, and molecular docking were performed on these ferroptosis-related feature genes.

## 2. Materials and Methods

### 2.1. Data Collection and Processing

The TCGA-ESCA expression profile data, variation data, clinical information, and follow-up information were downloaded from the XENA database, and the samples of adults older than 18 years were screened. GSE161533 and GSE44021 [17] expression data and sample information were downloaded from GEO database. For data preprocessing, probes were mapped to genes according to the annotation file, and empty probes were removed. When multiple probes correspond to the same gene, the maximum value is selected as the expression level of the gene. The genes with low expression were filtered out according to the gene expression level greater than 1 in at least 10% of the samples.

### 2.2. Ferroptosis Gene Set Selection

The ferroptosis-related gene signature in research of cancers [18, 19], and the databases of FerrDb [20], MsigDB [21], GeneCards [22] and KEGG [23], a ferroptosis gene set are integrated. Furthermore, we conducted correlation analysis between these genes and the normalized expression data of the TCGA-ESCA dataset, and screened the genes with Pearson's correlation coefficient greater than 0.6 and a *P* value less than 1*e* − 10 as ferroptosis candidate genes.

### 2.3. Differentially Expressed Genes Analysis

Differentially expressed gene analysis was performed on the FPKM normalized data in TCGA-ESCA using the limma package [24] in R. The |fold change| >1.2 and *P* value <0.05 were used as the thresholds for screening differentially expressed genes (DEGs) between tumor and normal tissues.

### 2.4. Cox Regression Analysis

The coxph function of the survival package [25] was used for univariate Cox analysis of individual genes or clinical characteristics (stage, gender, and age). *P* < 0.05 was used as the threshold to screen prognostic genes or clinical characteristics. After extracting the corresponding modeling parameters, the forest map was drawn using the forestplot package [26]. Then, the obtained genes or clinical characteristics were subjected to multivariate Cox analysis using the coxph function of the survival package.

### 2.5. Survival Analysis

Genes related to survival analysis were screened and grouped by the surv_cutpoint function in the survminer package [27] or expression median according to the expression level. Survival information and grouping information were fitted by the survfit function of the survival package. Finally, ggsurvplot function in the survminer package was used for analysis and visualization.

### 2.6. Feature Factor Screening by Random Forest

The random forest was used to further screen potential ferroptosis factors. The carat package was used to build a three-fold 10-fold crossover model, and random forest package [28] was used for analysis according to the optimized parameters. Finally, the genes with top 10 MeanDecreaseGini scores were selected as ferroptosis feature genes. Based on these feature genes, tumor samples were used to perform prognostic evaluation and ROC curves were plotted using the pROC package [29].

### 2.7. Gene Set Variation Analysis (GSVA)

The expression values of the ferroptosis feature genes were obtained from the normalized expression data of TCGA-ESCA, and the GSVA package [30] was used for GSVA analysis of the normalized expression data to obtain the complex values.

### 2.8. Prognostic Complex Survival Prediction

The screened ferroptosis genes were constructed as the GSVA set. The GSVA package was used for GSVA analysis of tumor samples to obtain the complex value. The coxph function of the survival package [25] was used to conduct regression modeling for the complex value, and the surv_cutpoint function was used to divide the samples into high- and low-risk groups according to the complex value, and the progression-free interval was analyzed.

### 2.9. Radiotherapy Effect Analysis

The TCGA-ESCA radiotherapy data were downloaded using the TCGAbiolinks package [31]. The complex score of these cases were divided into high and low complex value groups using the surv_cutpoint function, and then, the radiotherapy effect was statistically analyzed.

### 2.10. Independence Verification of the Complex Value and Nomogram Construction

In order to verify the independent prognostic efficacy of the complex value, univariate Cox analysis was performed on the TCGA-ESCA dataset by combining clinicopathological features (stage, gender, age, and complex value). Then, multivariate Cox regression was used to analyze the overall prognosis of the abovementioned four factors (stage, gender, age, and complex value) to verify the independent prognostic effect of the risk score. The cph function in the R package RMS [32] was used to construct the Cox proportional risk regression model, and the survival package was used to calculate the survival probability. Finally, a nomogram was constructed using the nomogram function and a correction curve was drawn to evaluate the prediction accuracy of the nomogram.

### 2.11. Immune Cell Infiltration Analysis

The immune cell infiltration score file of TCGA was downloaded from the TIMER2 database [33], and the data related to TCGA-ESCA samples were screened. The CIBERSORT [34] score data were used to compare the differences of immune cells in samples with different tumor survival times, and the *t*-test in the R package rstatix [35] was used to analyze the differences and calculate Pearson's correlation between the complex value and the proportion of immune cells. In addition, the normalized expression data of GSE161533 and GSE44021 were used for the immune infiltration score analysis using TIMER2, followed by the difference analysis of immune cells.

### 2.12. Gene Set Enrichment Analysis (GSEA)

The key ferroptosis genes were analyzed with GO biological process (BP) and KEGG pathway enrichment analyses using the ClusterProfiler package [36]. Additionally, GO BP enrichment analysis was further conducted using GSEA [37]. For the same biological processes enriched in the two steps, similarity calculation and hclust clustering were performed using the GOSemSim package [38].

### 2.13. Correlation Analysis of Key Genes and Clinical Characteristics

The esophageal tumor location, lymph node metastasis, radiographic evidence and age in tumor samples, and the expression levels of the key ferroptosis genes were selected for mosaic correlation analysis using the vcd package [39].

### 2.14. Mutation Analysis

TCGA-ESCA Mutect2 mutation files were downloaded using the R package TCGAbiolinks [31], followed by the visualization of mutation types using the R package maftools.

### 2.15. Molecular Docking

The corresponding compound structure information was downloaded from the DrugBank database [40] and screened according to Lipinski's rule (hydrogen bond receptor ≤10, hydrogen bond donor ≤5, rotatable bonds ≤10, log value of lipo-hydro partition coefficient ≤5, molecular weight 180–480, and polar surface area ≤140). A total of 5464 small molecule compounds were obtained. The 3D structural information of the protein encoded by the tumor characteristic ferroptosis genes was searched in the PDB database [41]. The relevant structural information of BCAP31 was found, and the corresponding PDB file 4JZL was downloaded. After the related parameters of AutoDock Vina were set, AutoDock Vina [42] was used to dock with small molecular compounds, and the interaction force analysis was performed using the PLIP website [43]. The result was demonstrated with Pymol.

## 3. Results

### 3.1. Data Acquisition

The TCGA-ESCA dataset included 161 tumor and 11 normal samples; the GSE161533 dataset included 28 tumor and 28 normal samples; the GSE44021 dataset contained 73 tumor and 73 normal samples. By integrating the ferroptosis pathway genes in the literature [18, 19], and the databases of FerrDb, MsigDB, GeneCards, and KEGG, a ferroptosis gene set containing 292 genes was obtained. After correlation analysis of these genes and the normalized expression data of the TCGA-ESCA dataset, 4192 ferroptosis candidate genes were screened with Pearson's correlation coefficient greater than 0.6 and *P* value less than 1*e* − 10. After integration, 4484 genes were finally obtained.

### 3.2. Principal Component Analysis (PCA) and Differential Expression Analysis

**Figure 1:**
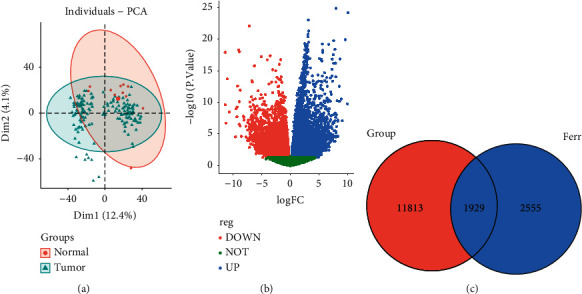
(a) Principal component analysis of the TCGA-ESCA dataset. (b) The Volcano plot of differentially expressed genes between ESCA and normal samples in TCGA. (c) Venn diagram of the intersection of differential genes and ferroptosis-related genes.

The FPKM expression data of TCGA-ESCA were processed with the limma package, and low expression genes were filtered and standardized, followed by PCA analysis, which showed significant differences between tumor and normal tissues (Figure 1(a)).

Differential expression analysis revealed that there were 7610 upregulated genes and 6132 down-egulated genes in the ESCA samples compared with the normal samples (Figure 1(b)). Venn analysis showed that there were 1929 intersection genes between the DEGs and the ferroptosis candidate genes (Figure 1(c)).

### 3.3. Univariate Cox Regression for Prognostic Factors Screening

**Figure 2:**
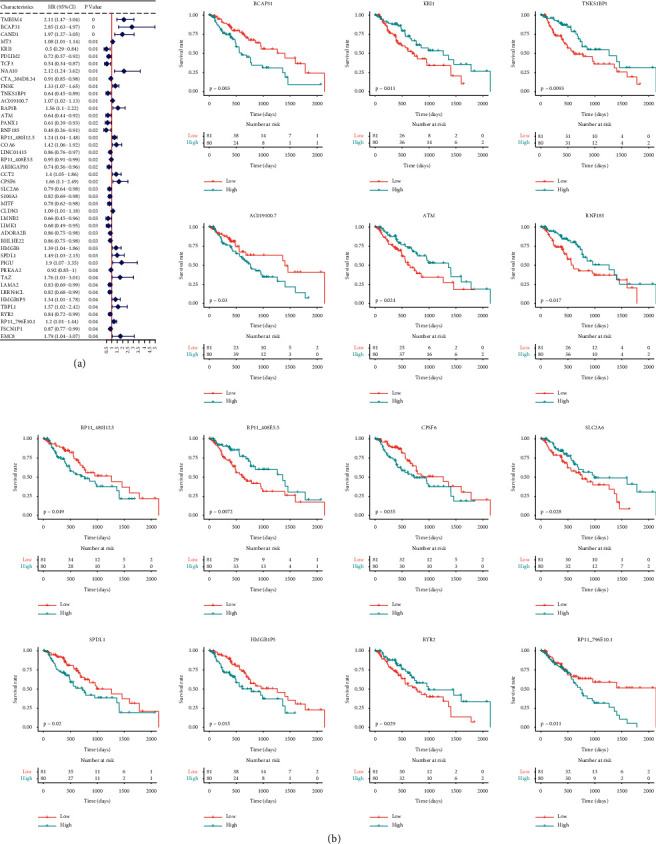
(a) The forest map of 44 prognostic genes. (b) The survival curves of 14 ferroptosis-related genes.

The tumor samples in the TCGA-ESCA dataset were screened and univariate cox regression analysis was performed on 1929 ferroptosis candidate genes. The results showed that 44 genes were significantly associated with prognosis of ESCA. The forest map of the prognostic genes is shown in Figure 2(a). Tumor OS analysis was performed on these 44 genes. Using the gene expression median as a threshold, the samples were classified into high-expression and low-expression groups. The expression levels of 14 genes were significantly correlated with the survival of ESCA (Figure 2(b)), which were considered as potential ferroptosis factors.

### 3.4. Further Screening of Ferroptosis Key Genes by Random Forest

**Figure 3:**
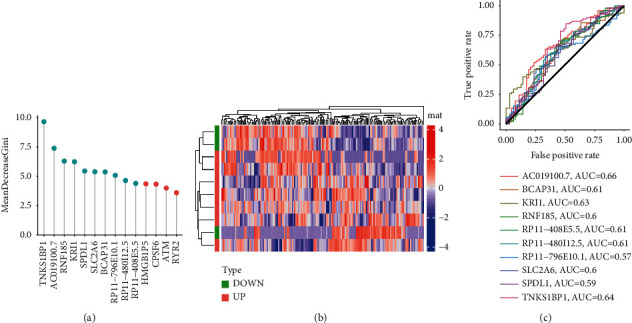
(a) MeanDecreaseGini scores of 14 potential ferroptosis factors. The blue node indicates key genes. (b) The expression level heatmap of ferroptosis key genes. (c) The ROC tumor prognosis curve of ferroptosis key genes.

The potential ferroptosis factors were further screened by random forest. Genes with the top 10 MeanDecreaseGini scores were selected as ferroptosis key genes, including *TNKS1BP1*, *AC019100.7*, *RNF185*, *KRI1*, *SPDL1*, *SLC2A6*, *BCAP31*, *RP11-796E10.1*, *RPL11-480I12.5*, and *RP11-408E5.5* (Figure 3(a)). The heatmap of the expression level of these genes is shown in Figure 3(b). The results of survival analysis of ESCA samples for these ten genes are shown in Figure 3(c). The ROC curves of each gene to analyze the tumor prognosis are shown in Figure 3.

### 3.5. Construction of a Prognostic Complex Value Based on the Key Genes

**Figure 4:**
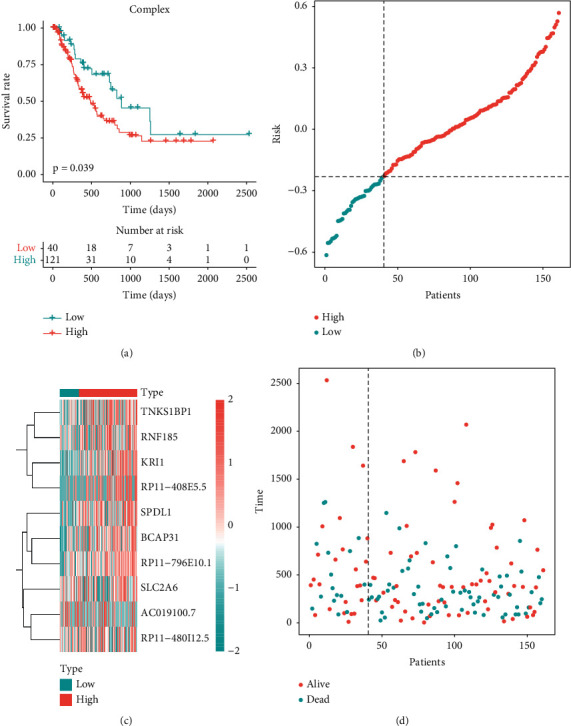
(a) There were significant differences in PFS between groups with high- and low-complex values. (b) The risk distribution of each group. (c) The correlation heatmap between the key gene expression levels and high/low complex values. (d) The survival prognosis time distribution of each group.

The ten ferroptosis key genes were constructed as the GSVA defined set. There were significant differences in PFS between groups with high-and low-complex values (Figure 4(a)). Risk distribution and survival prognosis time distribution of each group are shown in Figures 4(b) and 4(d). The correlation heatmap between the key gene expression levels and high/low complex values is shown in Figure 4(c).

Based on the TCGA-ESCA radiotherapy data, the radiotherapy effect of patients with a low complex score was better than that of high complex score. But there was no significance most likely due to the small sample size (Figure S1).

### 3.6. Construction of a Nomogram

**Figure 5:**
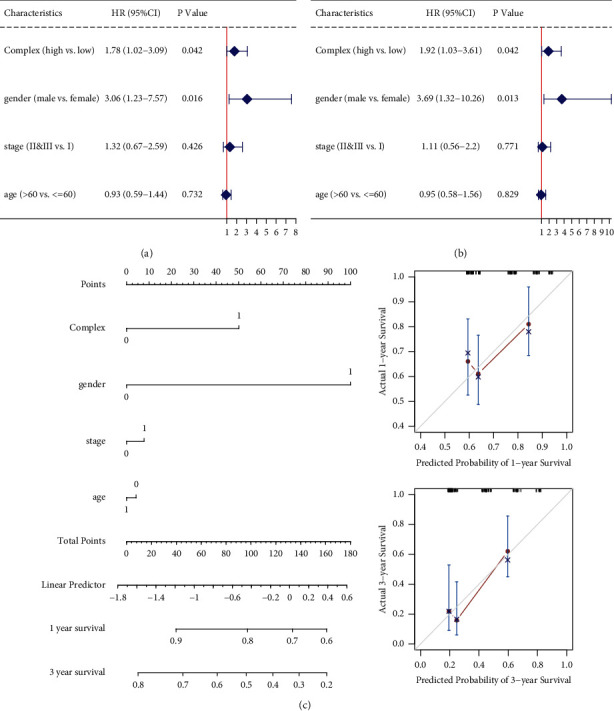
(a, b) Univariate (a) and multivariate (b) Cox regression analysis results. (c) Nomogram and calibration curves for one-and three-year survival.

Univariate Cox analysis was performed on the TCGA-ESCA dataset in combination with tumor stage, gender, age, and progression-free interval (PFI) survival data, as shown in Figure 5(a). Multivariate Cox regression analysis suggested that the complex value was an independent prognostic factor (Figure 5(b)). Then, the nomogram and calibration curves for one- and three-year survival were drawn (Figure 5(c)).

### 3.7. Correlation Analysis of the Complex Value and Immune Cells Infiltration

**Figure 6:**
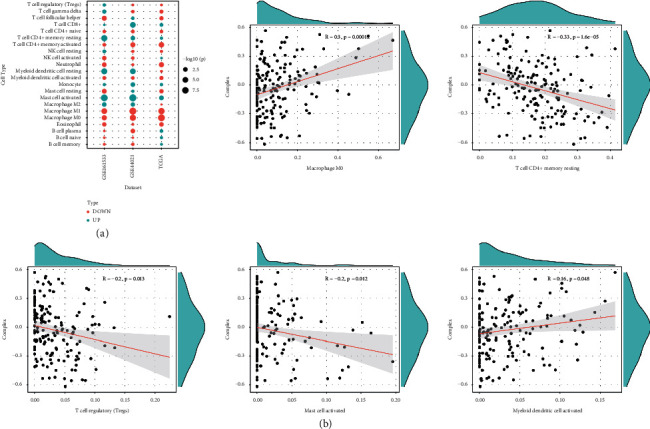
(a) The immune cell distribution differences in TCGA-ESCA, GSE161533, and GSE44021 datasets. (b) The correlation analysis between the complex value and the top 5 high proportions of immune cells.

The immune cell distribution differences in TCGA-ESCA, GSE161533, and GSE44021 datasets are shown in Figure 6(a). It was found that there was similar immune cell distribution between groups with high- and low-complex values in different ESCA datasets. The correlation analysis between the complex value and the top 5 high proportions of immune cells (macrophage M0, T cell CD4+ memory resting, T cell regulatory (Tregs), mast cell activated, and myeloid dendritic cell activated) is shown in Figure 6(b).

### 3.8. Functional Analysis of Ferroptosis Key Genes

**Figure 7:**
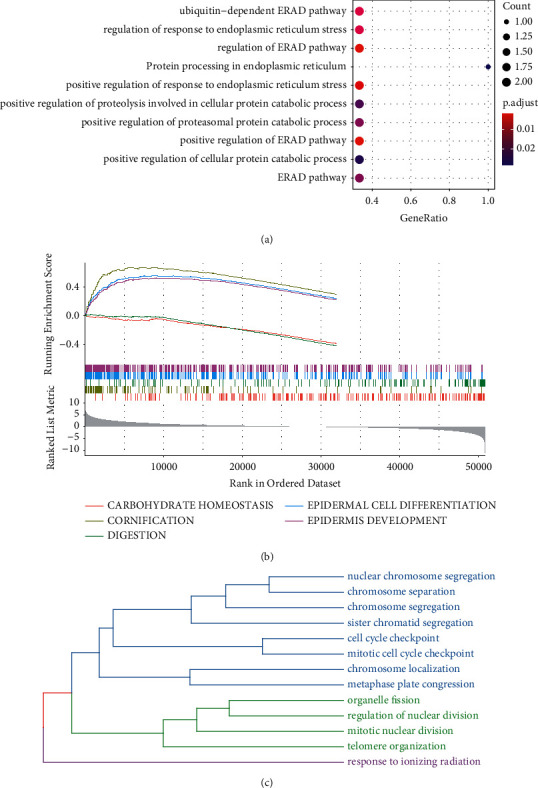
(a) Bubble maps of GO BP and KEGG pathways enriched by ferroptosis key genes. (b) The top five pathways of TCGA-ESCA sample enrichment analyzed by GSEA. (c) The same BP obtained from clusterProfiler and GSEA were mainly classified into three categories.

The ten ferroptosis key genes were significantly enriched in the ubiquitin-dependent ER-associated degradation (ERAD) pathway, regulation of response to endoplasmic reticulum stress, and regulation of the ERAD pathway based on the clusterProfiler package (Figure 7(a)). By using GSEA, GO, and BP terms of carbohydrate homeostasis, cornification, digestion, epidermal cell differentiation, and epidermis development were identified (Figure 7(b)). For the same BP obtained from clusterProfiler and GSEA, they were mainly classified into three categories (Figure 7(c)), suggesting that these genes were involved in similar BP.

### 3.9. Correlation Analysis between Key Genes and Clinical Features

**Figure 8:**
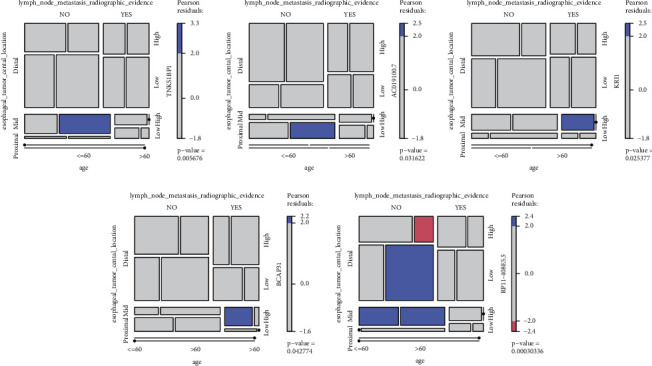
*TNKS1BP1*, *AC019100.7*, *KRI1*, *BCAP31*, and *RP11-408E5.5* were found to be significantly correlated with esophageal tumor central location, lymph node metastasis radiographic evidence, and age.

The *TNKS1BP1*, *AC019100.7*, *KRI1*, *BCAP31*, and *RP11-408E5.5* genes were found to be significantly correlated with esophageal tumor location, radiographic evidence of lymph node metastasis, and age. The esophageal tumor location was distal in most cases, and a small number of samples presented radiographic evidence of lymph node metastasis. High expression of *KRI1* and *BCAP31* predisposed to lymph node metastasis in patients under 60 years of age. Low expression of *RP11-408E5.5* reduced lymph node metastasis in patients with distal esophageal tumor location. In patients over 60 years of age with high expression of *TNKS1BP1* and low expression of *AC019100.7*, their esophageal tumor location in the middle part was less prone to lymph node metastasis (Figure 8).

### 3.10. Mutation Analysis of Key Genes

There was a high incidence of missense mutation in the key genes (Figure S2A). Specifically, missense mutation and frame shift mutation accounted for 43% in *KRI1*, missense mutation and splice site accounted for 29% in *RNF185*, and missense mutation accounted for 14% in *BCAP31*, *SLC2A6*, and *TNKS1BP1*. The point mutation type is shown in Figure S2B. The point mutation distribution of *KRI1* is shown in Figure S2C.

### 3.11. Screening of Potential Therapeutic Compounds Based on Molecular Docking

**Figure 9:**
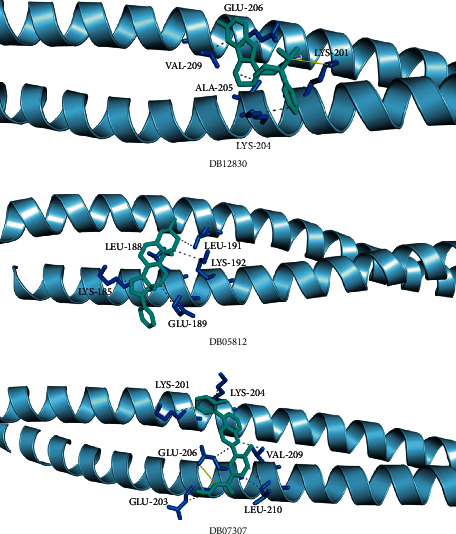
Conformation and interaction force analysis of BCAP31 with DB12830, DB05812, and DB07307. Cyan compounds are small molecules, yellow solid lines are hydrogen bonds, gray dotted lines are hydrophobic interactions, and blue amino acid residues that form hydrogen bonds with small molecules.

The top three small molecule compounds with the strongest binding ability for *BCAP31* were DB12830, DB05812, and DB07307 (Figure 9). The top 10 compounds with the highest docking score with *BCAP31* are shown in Table 1.

## 4. Discussion

Selective induction of cancer cell death is presently the most effective anticancer treatment [44, 45]. There are accumulating evidence showing that ferroptosis, a type of programmed cell death identified in recent years, plays a crucial role in tumorigenesis and the efficacy of cancer treatment [9, 45, 46]. Therefore, analysis of ferroptosis-related genes in ESCA may help identify novel biomarkers for prognosis and targeted therapy.

In this study, we focused on ferroptosis-related genes and investigated their influence on the prognosis of ESCA. Based on the TCGA dataset and the ferroptosis pathway genes obtained from the literature and public databases, 1929 ferroptosis-related genes were preliminarily selected. Following univariate Cox regression analysis and KM survival analysis, 14 genes were screened as ferroptosis-related factors. Then, random forest analysis further screened 10 ferroptosis key genes, which were used to construct a prognostic complex value. The low-complex value group indicated a better prognosis and radiotherapy effect compared with the high-complex value group. Further univariate and multivariate Cox regression analyses revealed that the complex value was an independent prognostic factor. Additionally, in different ESCA datasets, there were similar differences in the proportion of immune cell distribution between high- and low-complex value groups. Furthermore, *TNKS1BP1*, *AC019100.7*, *KRI1*, *BCAP31*, and *RP11-408E5.5* genes were significantly correlated with ESCA tumor location, lymph node metastasis, and patient age. Mutation analysis revealed that *KRI1* had the highest mutation frequency. Molecular docking results showed that *BCAP31* had the strongest binding ability with small molecules DB12830, DB05812, and DB07307.

Based on a series of analyses, ten ferroptosis key genes were identified and used to construct a prognostic complex value. The low-complex value group indicated a significantly better prognosis compared with the high-complex value group. Additionally, the complex value was an independent prognostic factor. The results suggested that these ferroptosis-related genes may serve as a prognostic signature of ESCA. Radiotherapy has an indispensable role in the management of ESCA. In recent years, radiotherapy has achieved a better balance between improving treatment efficacy and reducing toxicity [47]. In the present study, we analyzed the radiotherapy effect between the low- and high-complex value groups and found that the radiotherapy effect was better in the low-complex group, which was inconsistent with the results mentioned above that the low complex value group had a significantly better prognosis.

The protumor or antitumor state of immune cell infiltration in cancers is crucial for the efficacy of cancer treatment [48]. Recently, immunotherapy with immune checkpoint inhibitors has completely reversed the traditional treatment of ESCA [49]. In this study, the complex value was positively correlated with macrophage M0, and myeloid dendritic cell activated while negatively correlated with T cell CD4+ memory resting, T cell regulatory (Tregs), and mast cell activated. A recent study reported a prognostic model established by immune genes associated with memory CD4+ T cells, follicular helper cells, and monocytes for patients with ESCA [50], which was inconsistent with our results.

It was reported that the prognosis of ESCA depends on the extent of the primary tumor and lymph node metastasis [51]. Lymph node status is the most important prognostic factor, and an increased number of metastatic lymph nodes are related to a poor prognosis in ESCA [52]. Five ferroptosis-related genes (*TNKS1BP1*, *AC019100.7*, *KRI1*, *BCAP31*, and *RP11-408E5.5*) were found to be significantly correlated with radiographic evidence of lymph node metastasis. TNKS1BP1 is a tankyrase-binding protein, which interacts with the actin-capping proteins and involves in cell motility and invasion in cancers [53]. BCAP31 is located on the endoplasmic reticulum membrane and involves in the crosstalk between the endoplasmic reticulum and the mitochondria to regulate apoptosis [54, 55]. It has been suggested that BCAP31 regulates the migration and invasion ability of cancer cells by regulating the expressions of cytoskeletal proteins [56, 57]. The roles of the other three genes in cancers have rarely been reported based on the best of our knowledge.

Mutation is one of the important factors leading to gene dysfunction [58]. It has been suggested that *TP53* is the most significantly mutated genes in ESCC with a mutation frequency reaching 93% [59]. *KRI1* is an ortholog of *KRIT1*, and *KRIT1* mutation has been identified in cerebral cavernous malformations [60]. Additionally, *KRIT1* was recently reported to control the progression of melanoma by acting as a tumor suppressor, suggesting the role of *KRIT1* in human cancer [61]. To our best knowledge, there has been no report about *KRI1* gene mutation in patients with ESCA. Given that *KRI1* had the highest mutation frequency in ESCA, we speculated that *KRI1* may play a critical role in ESCA.

Finally, the molecular docking results showed that *BCAP31* had the strongest binding ability with small molecules DB12830, DB05812, and DB07307, which suggested that DB12830, DB05812, and DB07307 may serve as candidate agents for the treatment of ESCA by targeting *BCAP31*.

In conclusion, we constructed a novel ferroptosis-related gene signature, which has the potential to predict the survival and tumor-infiltrating immune cells of ESCA. This gene signature could have a promising value in the individualized treatment of patients with ESCA.

## Figures and Tables

**Table 1:** The top 10 compounds with the highest docking score with *BCAP31*.

DrugBank_ID	Hydrogen acceptors	Hydrogen donors	Rotatable bonds	Log*P*	Molecular weight	TPSA	Affinity (kcal/mol)
DB12830	3	1	2	4.3	378.5	28.3	−7.9
DB05812	2	1	1	4.6	349.5	33.1	−7.8
DB07307	3	1	4	4.9	393.5	54.9	−7.8
DB12886	5	2	5	4.9	402.4	53.6	−7.7
DB08683	3	1	0	3.8	393.4	65.3	−7.6
DB00717	2	1	1	3	298.4	37.3	−7.5
DB02473	3	3	4	3.7	384.5	91.3	−7.5
DB07700	3	1	1	4.9	395.5	69.4	−7.5
DB01395	3	0	0	3.5	366.5	43.4	−7.4
DB03476	1	2	3	4.3	286.4	49.9	−7.4

## Data Availability

The data used to support the results are available at the TCGA (https://tcga-data.nci.nih.gov/tcga/) and GEO (https://www.ncbi.nlm.nih.gov/geo).

## Supplementary Materials

Supplementary Materials Figure S1: radiotherapy effects of different complex scores. Figure S2 A: the Waterfall plot of point mutation analysis of the key ferroptosis factors in tumor samples in the TCGA-ESCA dataset. B: a statistical diagram of the point mutation types. C: the point mutation distribution of KRI1.
