# Supplementary material for: A Novel Ferroptosis-Related Gene Signature to Predict Prognosis of Esophageal Carcinoma
Source: J Oncol. 2022 Jul 1;2022:7485435. doi: 10.1155/2022/7485435 (PMC9270146; doi:10.1155/2022/7485435)
Supplement: Supplementary Materials — Figure S1: radiotherapy effects of different complex scores. Figure S2 A: the Waterfall plot of point mutation analysis of the key ferroptosis factors in tumor samples in the TCGA-ESCA dataset. B: a statistical diagram of the point mutation types. C: the point mutation distribution of KRI1. [file 7485435.f1.docx]

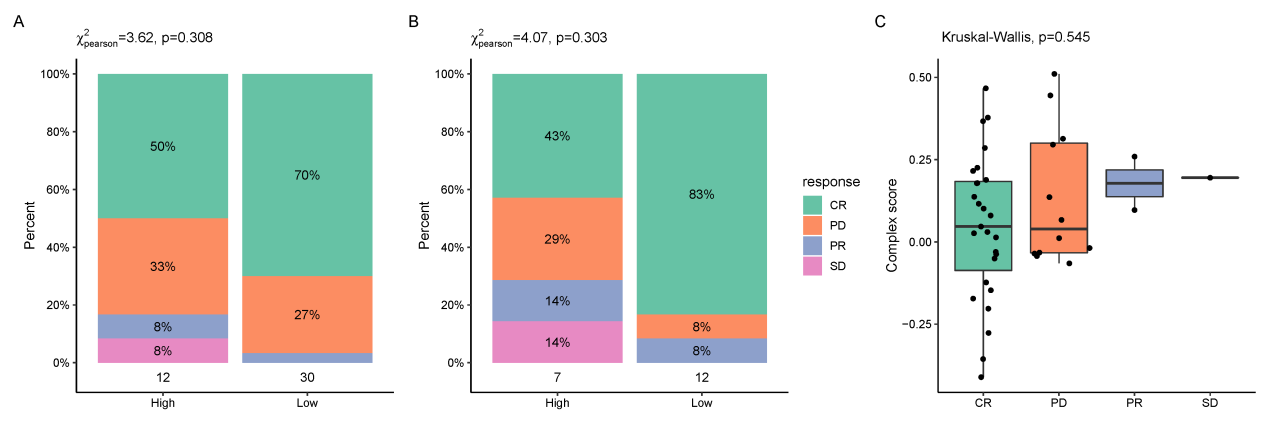


Figure S1 Radiotherapy effects of different complex scores.


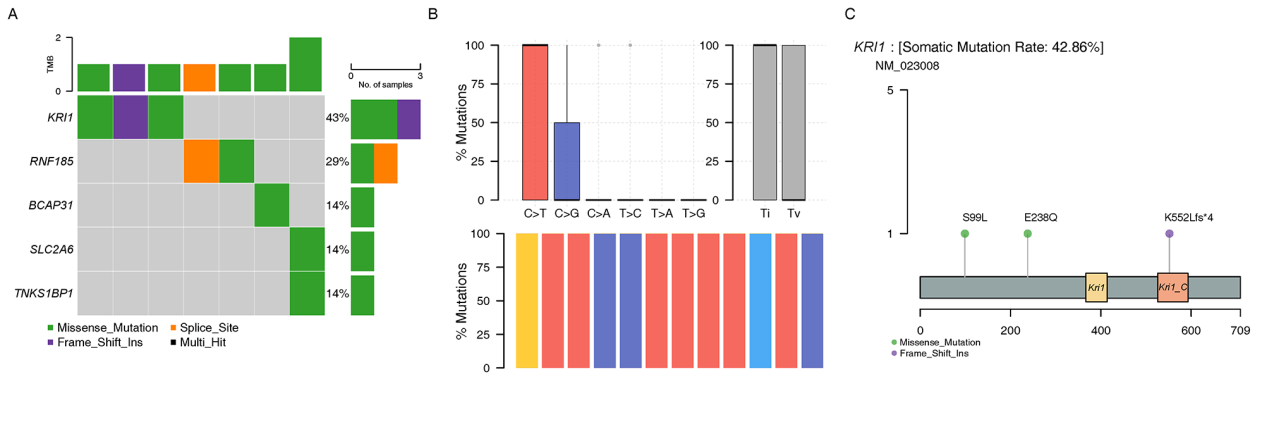


Figure S2 A: Waterfall plot of point mutation analysis of key ferroptosis factors in tumor samples in the TCGA-ESCA dataset. B: Statistical diagram of point mutation types. C: Point mutation distribution of KRI1.
